# Eye on the B-ALL: B-cell receptor repertoires reveal persistence of numerous B-lymphoblastic leukemia subclones from diagnosis to relapse

**DOI:** 10.1038/leu.2016.142

**Published:** 2016-06-17

**Authors:** R J M Bashford-Rogers, K A Nicolaou, J Bartram, N J Goulden, L Loizou, L Koumas, J Chi, M Hubank, P Kellam, P A Costeas, G S Vassiliou

**Affiliations:** 1 Wellcome Trust Sanger Institute, Wellcome Trust Genome Campus, Hinxton, Cambridge, UK; 2 Department of Medicine, University of Cambridge, Cambridge Biomedical Campus, Cambridge, UK; 3 The Center for the Study of Hematological Malignancies, Nicosia, Cyprus; 4 Department of Haematology, Great Ormond Street Hospital for Children, London, UK; 5 Genetics and Genomic Medicine Programme, UCL Institute of Child Health, London, UK; 6 Pediatric Oncology/Hematology Clinic, Nicosia, Cyprus; 7 Division of Infection and Immunity, Research Department of Infection, University College London, London, UK; 8 Cambridge Blood and Stem Cell Biobank and Cancer Molecular Diagnosis Laboratory, Cambridge Biomedical Research Centre, Cambridge, UK

## Abstract

The strongest predictor of relapse in B-cell acute lymphoblastic leukemia (B-ALL) is the level of persistence of tumor cells after initial therapy. The high mutation rate of the B-cell receptor (BCR) locus allows high-resolution tracking of the architecture, evolution and clonal dynamics of B-ALL. Using longitudinal BCR repertoire sequencing, we find that the BCR undergoes an unexpectedly high level of clonal diversification in B-ALL cells through both somatic hypermutation and secondary rearrangements, which can be used for tracking the subclonal composition of the disease and detect minimal residual disease with unprecedented sensitivity. We go on to investigate clonal dynamics of B-ALL using BCR phylogenetic analyses of paired diagnosis-relapse samples and find that large numbers of small leukemic subclones present at diagnosis re-emerge at relapse alongside a dominant clone. Our findings suggest that in all informative relapsed patients, the survival of large numbers of clonogenic cells beyond initial chemotherapy is a surrogate for inherent partial chemoresistance or inadequate therapy, providing an increased opportunity for subsequent emergence of fully resistant clones. These results frame early cytoreduction as an important determinant of long-term outcome.

## Introduction

Advances in the treatment of B-cell acute lymphoblastic leukemia (B-ALL) have increased long-term survival of pediatric patients to above 80%, although the equivalent rate for adults remains poor at 30–40%,^1, 2^ with relapse representing the leading cause of mortality at all ages. B-ALL is thought to arise from the leukemic transformation of a lymphoid precursor at an early stage of B-cell differentiation. B-cells express distinct cell-surface B-cell receptors (BCRs), generated during B-cell differentiation through the rearrangement and assembly of heavy- and light-chain gene variable (V), diverse (D) and joining (J) elements into V(D)J segments through V(D)J recombination.^3^ BCRs represent unique markers for each B-cell clone, where the accumulation of BCR mutational variants have been reported to occur at significantly greater rates than that of the rest of the genome,^4, 5, 6^ making this genomic region ideal for characterization of B-cell population dynamics by high-throughput sequencing.^7, 8^ The BCR sequence repertoire of an individual thus represents a snapshot of their B-cell population structure and can identify the presence of clonal proliferations, making it useful in the diagnosis and monitoring of B-cell malignancies.^9^ Next-generation sequencing of BCR repertoires^10^ can therefore facilitate the longitudinal study of the clonal dynamics of malignant B-cell populations from diagnosis to relapse.

The early clonal dynamics of ALL during treatment are highly predictive of relapse^11, 12, 13^ as is the detection of minimal residual disease (MRD) at later stages^14^ in both children and adults,^7, 15, 16^ with most reported cases of B-ALL relapse associated with the acquisition of drug resistance mutations. Here, we develop a robust protocol for high-throughput sequencing and analysis of BCR sequence repertoires in B-ALL and demonstrate that it has equal or superior sensitivity and specificity for MRD detection compared with fusion gene qRT-PCR, when applied to the same RNA material used for the latter. We then use our platform to study DNA samples in the same way in order to (a) decipher the clonal architecture of serial patient samples taken at diagnosis, during treatment and, where applicable, at relapse and (b) characterize the population dynamics between paired diagnosis and MRD-positive samples. We find multiple related malignant clone clusters, many of which persist from diagnosis to relapse, indicating partial chemoresistance and/or inadequate therapy in those who go on to relapse. Our findings support the premise that despite significant reductions in the number of bulk B-ALL cells with initial therapy, primary partial chemoresistance affords clonogenic leukemic cells with the opportunity to acquire additional *bona fide* resistance mutations culminating to relapse.

## Materials and methods

### Samples

Total nucleated bone marrow (BM) cells were isolated from aspirate samples after erythrocyte lysis and peripheral blood (PB) mononuclear cells from 10ml of blood after Ficoll and erythrocyte lysis. Total RNA was isolated and purified using QIAamp DNA/RNA blood mini-kit and QIAcube Automated Robotic System (Qiagen, Manchester, UK). Samples were derived from (i) patients with archived samples, which were studied using informative qRT-PCR for fusion genes and (ii) patients who went on to relapse after achieving remission.

### BCR amplification, sequencing and assembly

RT-PCR and PCRs were performed as described previously^8^ using FR1 primer(s). MiSeq libraries were generated and reads filtered as described previously (detail in Supplementary information).^10^ The network generation algorithm and network properties were calculated as in Bashford-Rogers *et al.*:^8^ each **vertex** represents a unique sequence, where relative vertex size is proportional to the number of identical reads. **Edges** join vertices that differ by single nucleotide non-indel differences and **clusters** are collections of related, connected vertices. Alignments were performed using Mafft^17^ and maximum parsimony trees fitted using Paup* version 4 using the best-fit nucleotide substitution model in MODELTEST.^18, 19^ Aligned sequences were then analyzed using a new computational pipeline, MRD Assessment and Retrieval Code in pYthon (MRDARCY), to identify all clusters ⩾2.5% in index samples, and to subsequently mine all sequences with ⩽8 bp mismatches from the clusters in all samples associated with the corresponding patient.

### Secondary rearrangement analysis

BCRs represented by >2 reads from all clusters greater than 2.5% of the total repertoire for each patient were captured and their stem regions identified (defined as *N-IgHD-N-IgHJ* regions starting 3bp downstream of the *IgHV* gene boundary). All BCRs containing these stem regions were captured at each time point or searched-for in unrelated healthy controls. Stem regions were clustered together by similarity (where all stem regions within a cluster are related to at least one other member by a single bp difference) and the relative frequencies of each *IgHV* gene, represented by sequences containing the stem sequence group, were determined by BLAST^20^ using the IMGT reference gene database.

### qPCR, blast quantification and cytogenetics

qPCR for fusion genes was performed as in *Gabert et al.*^21^ Blast counts were assessed from BM or PB (Supplementary Table S1) as a percentage of total nucleated cells by morphology.^22^ Complete remission was defined as BM blasts <5%. Day 28 MRD was performed by RT-PCR.^23^ Interphase nuclei were screened by fluorescence *in situ* hybridization to assess abnormal cytogenetics at diagnosis and relapse.^24^

### Mutational profile and PCR/sequencing error analysis

To test the significance of the overlap between diagnosis and relapse samples, where the null hypothesis assumed that the most frequently observed BCR sequences (the central BCRs in each tree) are the only BCRs retained throughout therapy and represent the progenitor cell(s) of all relapsed B-ALL cells. If the BCR sequence length is *l*, the nucleotide distance from the central BCR *d* and any position can mutate to any of three other bases, then the number of potential mutational combinations is:


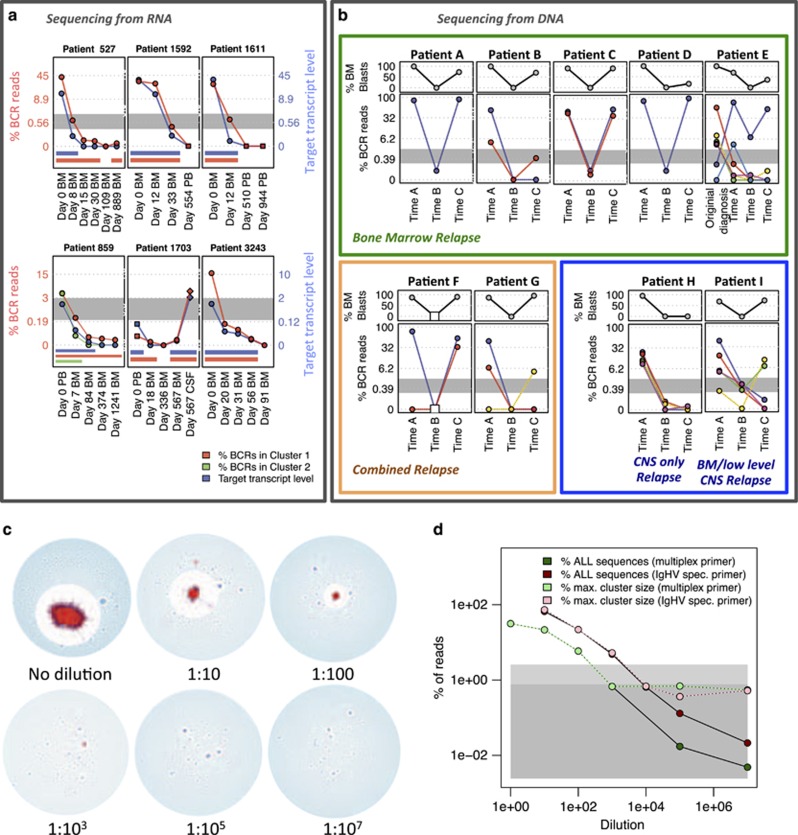


The hypergeometric test in R was used to calculate the probability of observing equal or greater BCR sequence overlap between diagnosis and relapse by chance for all pairs tested from each aligned B-ALL BCR sequence cluster. The same analyses were performed on the data sets for control genes (human *GAPDH*/β-globin from healthy donors in Bashford-Rogers *et al.*^8^). The 'true' control gene sequence within each control sample was defined as the highest observed sequence in the sample, where sequence variants from these were defined as PCR/sequencing errors. The probabilities of random overlap between the sites of PCR/sequencing error were calculated using the hypergeometric distribution (probability for observed or greater overlap to occur by chance) (Supplementary Figure S7).

To account for any potential sequencing/PCR mutational biases, the baseline observed probabilities of overlap between mutations in independent V-(D)-J recombination events were used to compare the diagnosis-relapse samples: for all the healthy control samples, we identified all sequences within clusters of more than six sequences, and mutations within the V genes were determined. By comparing the occurrence of each V gene mutation between independent clones (defined as either clones sharing the same V gene allele but a different J gene or same V gene allele in dependent healthy individuals), the baseline frequency of shared mutations was calculated. Similarly, the mutations away from the central BCR in the B-ALL clone between diagnosis and relapse samples from each relapsing patient were determined, and the distribution of overlapping mutational sites was determined. The difference in frequency of overlapping mutations between the healthy V gene independent mutations and the diagnosis-relapse mutations was statistically tested using the Mann–Whitney test (one-sided).

## Results

### B-ALL BCR analysis and identification of clonal sequences

Longitudinal samples (*n*=56) from 15 B-ALL patients taken over the course of therapy were analyzed for the presence of residual leukemia by qPCR for transcript levels of fusion genes (six patients, treated as per UKALL XI protocol) or by % BM blasts (nine patients, treated as per UKALL2003 protocol). The samples were studied by analysis of BCR sequencing repertoires and mining of leukemia-specific BCR sequences derived from RNA (UKALL2003 samples) or DNA (UKALL XI samples) (Table 1). The ‘primary' sample for each patient, defined as the one with the highest leukemic load, which was the diagnostic (pre-treatment) sample for all patients except 1703 (567 day relapse sample used) and patient 859 (undergoing therapy on day 0), was used to identify unique V(D)J sequences associated with the B-ALL clone(s) (Supplementary Table S1). Additionally, BCR sequencing was performed on PB from 18 healthy individuals aged 20–75 years. After filtering, network analysis^8^ was performed on BCR sequencing data verifying clonality in all B-ALL primary diagnostic samples (largest cluster sizes of 5.7–83.64% of the total BCR repertoire) and the day 567 sample from patient 1703 (largest cluster 3.83%) (Supplementary Table S1). By comparison, the largest clusters from the healthy individuals averaged 0.60% (standard deviation of 0.64%, range 0.14–2.577%, Supplementary Table S2). High reproducibility was observed between the network structures of two independent PCR amplification and sequencing runs (Supplementary Figure S1).

**Figure 1:**
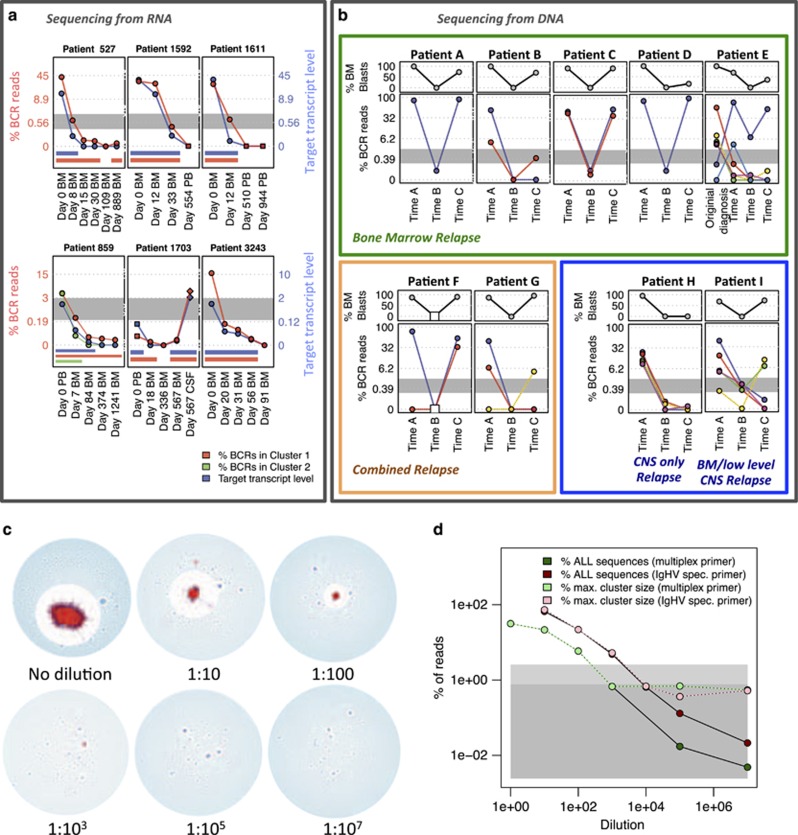
BCR sequencing: evaluation of sensitivity and detection of B-ALL clones. (**a**) qPCR target/control (T/C) transcript ratios (blue) and percentages of RNA-derived clonotypic B-ALL BCR reads over time for each patient (red for largest cluster and green for second largest cluster, where present). The blue axes (right of each plot) refer to the T/C qPCR transcript ratio levels and the red axes (left) to the percentage of sequences in the corresponding clusters (log2 scales). Blue and red bars under each plot indicate time points that are positive for qPCR transcripts and B-ALL BCR reads, respectively. The initial sample for patient 1703 was taken 2 weeks after starting treatment, hence the low levels of qPCR and clonotypic BCR positivity at time 0. BM, bone marrow; PB, peripheral blood; CSF, cerebrospinal fluid sample. (**b**) Variation of percentages from nine B-ALL patients of BM blasts (top panels) and percentages of DNA-derived clonotypic B-ALL BCR reads over time (bottom panels, different colored lines are used for each individual clone larger than 2.50% of the total BCR repertoire at any of the indicated time points). Percentage of sequences in the corresponding clusters is plotted in a square scale to highlight lower frequency observations. Time points A, B and C refer to diagnosis, day 28 and relapse for each patient, and missing samples are indicated by white squares (patient F, time B). The gray-shaded area shows the maximum cluster sizes for healthy patients (mean±2s.d.). Patients are grouped by clinical relapse type, with BM relapse patients in the green box, combined relapse in the orange box and CNS only/predominant relapse in the blue box. (**c**, **d**) RNA from a B-ALL patient sample was mixed with RNA from healthy peripheral blood mononuclear cells (PBMCs) at different ratios. BCR sequencing was performed using the full set of multiplex primers or the single primer with the best alignment to the malignant B-ALL BCR sequence (*IgHV*-specific primer), each yielding an average of 125 642 filtered BCR sequences (range of 18 970–294 354). (**c**) Network diagrams showing sequential dilution of B-ALL into healthy blood RNA using the multiplex primers, where clusters within 8 bp sequence similarity to the B-ALL cluster are marked in red and all others in blue. (**d**) Percentages of BCR sequences corresponding to the B-ALL BCR population at each dilution using multiplex primers (dark-green) and *IgHV*-specific primer (dark-red). Overlaid is the percentage in the largest BCR cluster (irrespective of relationship to B-ALL) for multiplex primers (light-green) and IgHV-specific primer (light-red).

Using our bespoke computational pipeline (MRDARCY—see Materials and methods), we identified B-ALL clonotypic BCRs in the diagnostic sample and searched diluted or serial patient samples for identical or related BCRs, allowing for a set number of base-pair (bp) mismatches (⩽8 bp in this study). MRDARCY was used to identify clonotypic sequences (clusters representing ⩾2.5% of the entire repertoire, above the 95th percentile of the healthy range) in the primary samples from six patients treated on the UKALL2003 protocol and to determine the percentage of matching BCR sequences in longitudinal RNA samples, also studied for MRD using fusion gene qPCR. BCR sequencing concurred closely with qPCR transcript levels (red/green versus blue lines, Figure 1a), demonstrating strong correlations between the percentage of clonotypic B-ALL BCRs and qPCR T/C ratios (*R*^2^ values>0.87, Supplementary Table S3), while B-ALL clonotypic BCR sequences were detected in all qPCR-positive samples. Detection of very low-level B-ALL sequences in some qPCR-negative samples was reproducible in replicates of the same sample, with the exception of one sample (Supplementary Table S4, patient 1611, day 19 sample), from which clonotypic sequences were detected at low frequency (0.0656%) in only one of two repeats in keeping with stochastic ‘loss' of a rare variant.^8, 25, 26, 27^ Likewise, the percentage of matching BCR sequences in DNA samples (UKALL XI) correlated with the % blasts in PB (Figure 1b). Notably, we detected clonotypic sequences in 6 of 10 patients at day 28, where blasts were not detected (<1%). Therefore, our results show that BCR sequencing is highly sensitive as a method for MRD detection and at least as good as fusion gene qPCR.

### Sensitivity for detection of B-ALL clonotypic BCRs

To quantify the sensitivity of BCR sequencing, we performed a titration experiment using serial 10-fold dilutions of a known clonal B-ALL RNA sample (1592_A) into healthy PB RNA. With 31.41% of all BCR sequences in the undiluted sample related to the leukemic cluster, ALL clonotypic BCRs were detected in dilutions as low as 1 in 10^7^ healthy PB RNA molecules (Figures 1c and d). Notably, clonotypic BCR sequences from any of the B-ALLs studied were present by chance only at a rate of 1 in 2 720 172 unique BCR sequences from 8 healthy blood samples amplified in the same way (Supplementary Table S5). Sensitivity was further increased 13.57 × when the single primer (from the 6-primer multiplex set) that best amplified the leukemic BCRs was used (Figure 1d, Supplementary Table S6), suggesting that a semi-bespoke approach could make this an even more powerful MRD tool. Similarly for DNA, previous studies have demonstrated detection of clonotypic sequences down to at least 1:10 000 dilution.^28^

BCR RNA expression levels in mature B cells are higher than those in pre-B-cells or immature B cells,^29^ including B-ALL cells, and this could lead to underestimation of the fraction of malignant B cells in a sample. We have previously shown that there is a strong linear correlation in the frequencies of functional BCRs between RNA and DNA from the same sample.^27^ Here, by studying three patients with both DNA and RNA available from the same B-ALL sample, we find that DNA-amplified B-ALL BCR sequences represented a much higher percentage of total BCR sequences than RNA-derived ones (Supplementary Figure S2), most likely due to differences in RNA expression between B-cell subsets and differences in RNA stability the B-ALL clone compared with their healthy B-cell counterparts.^30, 31^ Therefore, the RNA BCR repertoire, although very powerful in detecting low-level MRD, appears to underestimate the absolute fraction of residual B-ALL cells in a sample. In support of this, 80% of cells in the day 567 cerebrospinal fluid (CSF) sample from patient 1703 were leukemic blasts (CD10^+^, CD19^+^, CD45^low/−^), while RNA-derived clonotypic B-ALL sequences represented only 3.38% of all BCR sequences (Supplementary Table S7). Therefore, the use of DNA has the potential to further increase the sensitivity of BCR repertoire analysis for MRD detection and to provide a better estimate of leukemic cell numbers as a percentage of total cells. Nevertheless, both nucleotide sources captured in detail the clonal architecture of B-ALL clones in each patient.

### Analysis of relapse cases: multi-clonal B-ALL from secondary rearrangements

**Figure 2:**
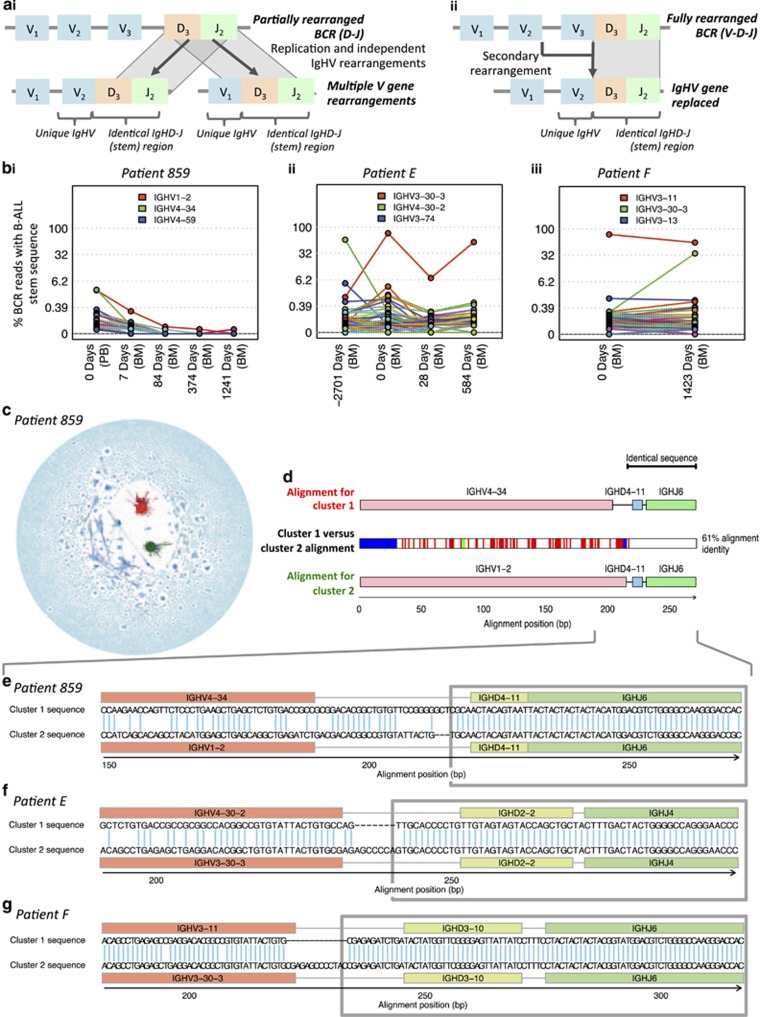
Secondary *IgHV* rearrangements in B-ALL subclones. (**a**) Schematic representation of different mechanisms of secondary *IgHV* rearrangements. (i) *Independent IgHV rearrangements*: After the D-J rearrangement, an early B cell divides and the resulting cells undergo independent *IgHV* rearrangements, while retaining a common *IgHD-J* stem sequence. (ii) *IgHV replacement*: an upstream *IgHV* gene is rearranged onto a pre-existing D-J rearrangement. (**b**) High-throughput detection of secondary rearrangements in B-ALL patient samples for (i) patient 859, (ii) patient E and (iii) patient F. The percentages of BCR sequences containing the stem sequences from the major clones in each patient were identified in serial time points (encompassing the *IgHD-IgHJ* region and non-template additions up to 3 bp 3' to the end of the *IgHV* gene, Supplementary Table S8). Different *IgHV* gene usages are plotted in different colors, and the highest three observed *IgHV* genes indicated above the plots. The gray lines indicate the top 99th percentile frequency of each stem sequence in 18 healthy individuals (0% for i–iii). (**c**) Network diagram for B-ALL patient 859 at day 0, with vertices within the largest cluster (cluster 1) in red, vertices within the second largest cluster (cluster 2) in green and all other vertices in blue. (**d**) BCR sequence alignment of the dominant sequences from the two dominant clusters in patient 859, cluster 1 and cluster 2 representing 2.81 and 2.89% of BCRs, respectively. The cluster 1 and 2 sequences were aligned to each other, and the positions of differences between sequences are indicated by the colored boxes in the corresponding positions in the middle row, using red for mismatches, green for gaps in cluster 1 BCR and blue for gaps in cluster 2 BCR. The cluster 1 and 2 sequences were 100% identical to the germline genes of (*IgHV4-34-IgHD4-11-IgHJ6*) and (*IgHV1-2-IgHD4-11-IgHJ6*), respectively, where the red, blue and green boxes for *IgHV*, D and J genes mark the gene boundaries respectively. (**e**–**g**) Alignments of the two largest BCR sequence clusters for patient 859 (**e**), patient E (**f**) and patient F (**g**). The alignments with the reference *IgHV* (highlighted in red), *IgHD* (highlighted in yellow) and *IgHJ* (highlighted in green) genes are indicated with dashes (-) denoting alignment gaps. The regions of the BCR sequence that are identical between the two clusters are highlighted in the gray boxes.

Biclonal and multi-clonal ALL cases have been detected in previous studies,^32, 33^ while recent work has shown that aberrant activity of recombination-activating genes promotes genomic rearrangements critical to B-ALL pathogenesis.^34, 35^ This raises the possibility that increased recombination-activating gene activity may also generate secondary *IgHV* rearrangements, such as V-gene replacement, within the B-ALL clone. As *IgHD-J* combinations (including junctional regions), known as ‘stem sequences' and are stable in instances of secondary rearrangements (Figure 2a),^23^ we developed a computational approach to detect B-ALL stem sequences with different IgVH gene usages in high-throughput sequencing data (Figure 2 and Supplementary Figure S3). By comparing the frequencies of these stem sequences in healthy individuals, we account for false-positive detection rates for each stem sequence (that is, the chance that the same stem sequence can be generated by chance in independent B cells). We report that secondary rearrangements are very common in B-ALL, with an average of 32.52 different *IgHV* genes combined with the stem sequence per B-ALL (range 9–59 *IgHV* genes: above 99th percentile for healthy individuals; Supplementary Figure S3 and Supplementary Spreadsheet 1). By determining the frequency of each stem sequence in unrelated B-ALL patients, our false detection rate was 9.245 × 10^−6^. While it is possible that these secondary rearrangements may represent pre-leukemic remnants of clonal diversification, examples of cases where the clones are clearly part of the leukemia were identified in patients 859, E and F, in which large subclones exhibited identical *IgHD-J*, but different V genes (Figure 2).

The two largest clusters in patient 859 (day 0) corresponded to different *IgHV* gene rearrangements (*IGHV4–34* vs *IGHV1–2*), but identical junction and *IgHD-J* region (55 bp, including 3 bp of non-template additions) (Figures 2c–e, red and green clusters respectively), thus consistent with a secondary rearrangement. These clusters displayed similar properties, including mean mutational distance from the dominant sequence in each cluster (2.281 bp and 2.135 bp respectively, Supplementary Table S8). The cluster 2 *IGHV* gene (*IGHV1–2*) was closer to the *IGHD/J* gene locus than the gene in cluster 1 (*IGHV4–34*), and so cluster 1 would have been a later secondary rearrangement. Interestingly, even though cluster 2 became undetectable from day 84 (Figure 1), cluster 1 was never fully eradicated over 1241 days, suggesting that cells with this BCR sequence were differentially affected by therapy.

Likewise, the dominant clone in the original patient E B-ALL sample (Figure 1b, red line) was different from the largest clone identified at first and second relapses (Figure 1b, purple line) more than 7.4 years later. The identical 57-bp region spanning the *IgHD-IgHJ* region including 10bp of non-template addition between the *IgHV* and *IgHD* genes (Figure 2f, Supplementary Table S9) confirms that B-ALL relapse arose from a minor subclone of the original disease. Interestingly, the dominant clone at original diagnosis was small at time A (0.145%), while the dominant clone at time A was small at the time of original diagnosis (0.129%) (Figure 1b). A third subclone in this patient also related by secondary rearrangement (turquoise line, *IGHV2-70-IGHD2-2-IGHJ4*, Figure 1b) was present at low frequency at diagnosis (0.00367%) and became the second most dominant at time A (3.491%), but is lost thereafter. Together, these data show that subclones related by secondary rearrangement can respond differently to therapy, despite being closely related.

Patient F also shows the presence of a single dominant clone (80.6%) at diagnosis (time A, Figure 2), while relapse shows two clones including the original clone (55.9%) and a novel clone (33.0%). These clones appear to be related by secondary rearrangement, with identical 84 bp region spanning the *IgHD-IgHJ* and 13 bp non-template region (Figure 2g), suggesting clonal evolution of the leukemia. Therefore, BCR sequencing can detect multiple disease subclones generated through genomic diversification and potentially even rare cases of two independent B-cell malignancies,^8, 36^ irrespective of their composition of driver mutations.

An additional consideration when BCR sequencing from DNA, rather than RNA, is that in some B cells initial BCR rearrangements are defective and followed by rearrangement of the second BCR (*IgH*) allele,^37^ something seen commonly in B-ALL clones.^38^ B-ALLs arising from such B cells would thus mimic the presence of two independent leukemias when DNA is used to amplify/sequence BCRs. However, bi-allelically rearranged B-ALLs should exhibit a strong correlation between sequencing frequencies of the two rearranged BCRs, whereas BCRs from independent clones would diverge over time. A bi-allelic rearrangement is likely to have occurred in the B-ALL from patient C (Table 1) for which only a single cytogenetic abnormality (*ETV6-RUNX1*), but two clonal BCR rearrangements were present and highly co-correlated through the disease course (*R*^2^ value=0.988).

### Association of relapse patterns with BCR repertoires

Differences in B-cell repertoires were associated with different patterns and sites of relapse. For all cases without cytogenetic evolution (9/10 of relapse patients), relapse was associated with re-emergence of B-ALL clones present at diagnosis (including clones related by secondary rearrangements). Patients A–E exhibited relapse in the BM only, where the dominant BCR clone(s) at diagnosis (time A) re-emerged at relapse (time C) (Figure 1b). Patient F shows the re-emergence of the original diagnostic clone at relapse as well as a novel clone related by secondary rearrangement as described earlier. The single case where cytogenetic clonal evolution occurred between diagnosis and relapse (patient G, additional gain of chromosome 21) corresponded to the emergence of a novel B-ALL BCR clone at relapse. Here, the original diagnostic B-ALL clonal BCR sequences were not detected at relapse. For patients 1703 and H, with CNS relapse (no blasts in BM), there were only low-level B-ALL BCRs detectable in BM DNA. In particular, only 0.353% of B-ALL BCRs was detected in BM DNA from patient H (Figure 1b) and for patient 1703 (RNA sample) the CSF was found to contain a significantly higher proportion of B-ALL BCRs compared with the BM (Figure 1a). These findings suggest that changes in BCR sequences may signify differences in biological behavior and that studying interim CSF samples for MRD may be of clinical value.

### B-ALL clonal complexity is significantly retained between diagnosis and relapse

**Figure 3:**
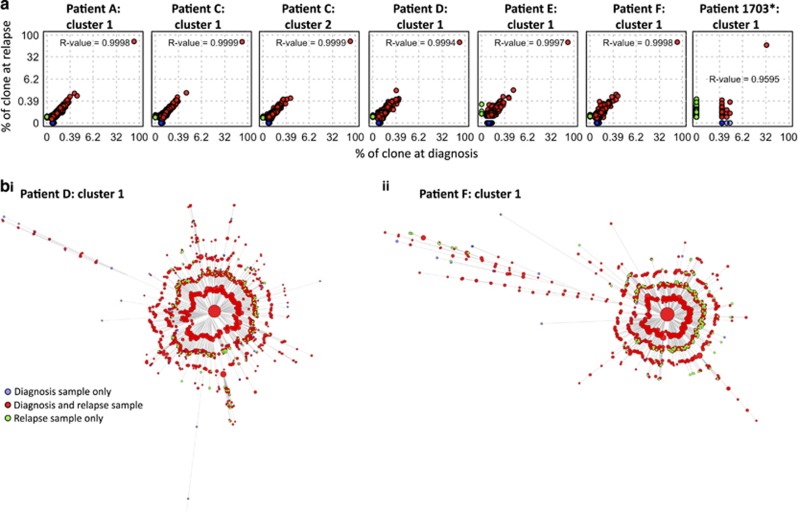
Phylogenetic analysis of paired diagnosis-relapse B-ALL samples. (**a**) The correlation of B-ALL BCR frequencies between diagnostic and relapse samples (as a percentage of reads in the corresponding clone) observed in (from left to right) DNA samples for patient A cluster 1, patient C cluster 1, patient C cluster 2, patient D cluster 1, patient E cluster 1 and patient F cluster 1 and for patient 1705 cluster 1 (day 0 (combined RNA and DNA sequencing data sets) against relapse (day 567, RNA sequencing data set)). Point colors are blue if the BCR was present only in the diagnostic sample, red if present in both the diagnostic and relapse samples, and green if present only in the relapse sample. Cube-root scales used to highlight the low-frequency BCRs, and presented with the corresponding *R*^2^ values. (**b**) Unrooted maximum parsimony trees showing the relationships between sequences observed in diagnostic and relapse DNA samples for (i) patient D cluster 1 and (ii) patient F cluster 1. Branch lengths are proportional to the number of varying bases (evolutionary distance). Bootstrapping was performed to evaluate the reproducibility of the trees suggesting strong support for the majority of the branches (>70% certainty for branches). Tips represent BCR sequences with point sizes correlating with the proportion of reads for a particular sequence (for display purposes this is not the case for the central cluster, whose size is fixed). The tip colors are blue if the BCR was present only in the diagnostic sample, red if present in both the diagnostic and relapse samples and green if present only in the relapse sample. *The day 0 sample for patient 1703 was taken after chemotherapy had started, hence the depletion of the diagnostic repertoire reflected in the dominance of green tips.

**Figure 4:**
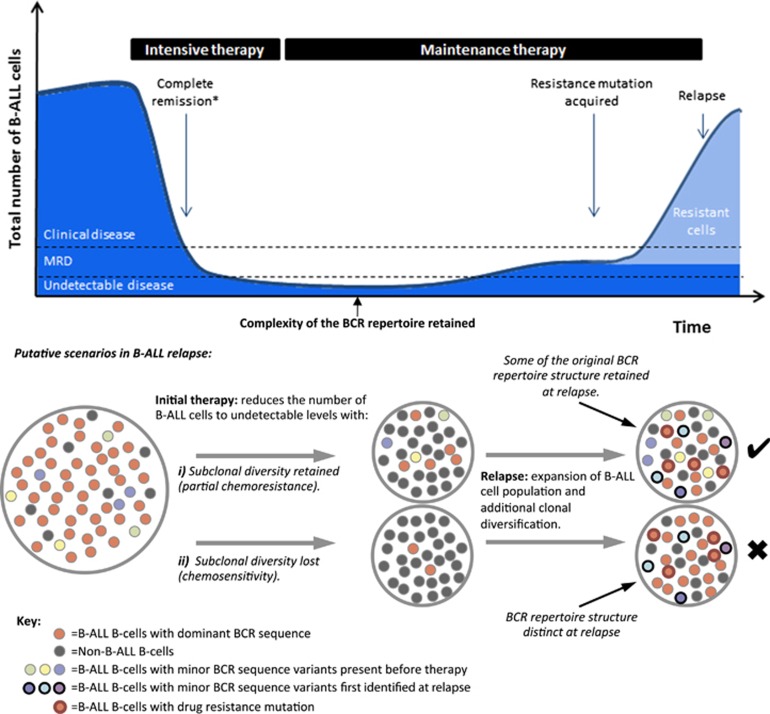
Proposed significance of the overlap between diagnostic and relapse BCR repertoires. Initial intensive therapy reduces the number of B-ALL B cells in a patient to MRD levels and then to undetectable levels before the start of maintenance therapy. During maintenance therapy, the likelihood of acquisition of resistance mutations is proportional to the number of residual clonogenic B-ALL cells. In the depicted example, multiple independent clonogenic cells survive intensive therapy because of relative inherent chemoresistance (or inadequate dosing) and persist throughout maintenance therapy. This increases the likelihood of and leads to the acquisition of a resistance mutation in one of these cells. Nevertheless, B-ALL remains suppressed and undetectable while on maintenance therapy only to relapse after treatment is completed (relapse while on still on treatment is evidently also possible through the same mechanism, but less common). While the main B-ALL clone dominates both the diagnosis and the relapse BCR repertoires, smaller related clones are detected at both these time points providing a surrogate for the inherent chemoresistance of the particular B-ALL or inadequate dosing for the individual patient. *No disease detectable by light microscopy.

Despite the fact that adenosine-induced deaminase (AID) expression is detected only in some B-ALLs,^39, 40, 41, 42^ the accumulation of low-level AID- or non-AID-mediated BCR mutations can result in clonal diversification.^43^ Indeed, the mutations within these clusters are enriched within the CDR1-3 regions compared with FWR1-3 regions (Supplementary Figure S4) and in AID mutational motifs (Supplementary Table S10), suggestive of AID activity.^44, 45, 46^ These mutations can be used to infer the hierarchical structure and extent of BCR diversification from a leukemia-initiating B-cell to bulk leukemia. To investigate the effect of treatment on the population structure of B-ALL, we studied diagnostic-relapse pairs exhibiting related BCRs, thus permitting sequence comparisons (patients A, C–F). B-ALL clonal sequences from diagnosis and relapse of each patient differing by ⩽8bp were aligned using Mafft^17^ and maximum parsimony trees fitted using Paup*.^18^ To reduce the risk of including sequences with errors, only BCRs that were observed at least twice were included (Figures 3a–f). The same analysis was performed on BM and CSF relapse samples from patient 1703 between day 0 (RNA and DNA data sets) and day 567 relapse (Figure 3g). The star-like structure of each tree reveals that the original B-ALL clone for each of these patients emerged from a single common ancestor,^47^ represented by the central BCR, which was in all cases the most frequently observed BCRs in both the diagnostic and relapse samples. Strikingly, beyond the central BCR, there was also high sequence overlap between the entire diagnostic and relapse sample BCR repertoires (>78% of unique BCRs within each clone for patients A–F and 13.6% for patient 1703, whose day 0 sample was collected after initial anti-leukemic therapy), even at distances of >7 nucleotides from the central BCR for each patient (Figure 3 and Supplementary Table S11). The strong linear correlations between the B-ALL BCR frequencies at diagnosis and relapse (*R*^2^ values>0.95, Figure 3) indicate that much of the population structure of the B-ALL clones is retained throughout the course of therapy and while the disease is in clinical remission (Figure 4).

The hypothesis that a *population* of B-ALL clones with distinct BCR sequences persisted through therapy and re-emerged at relapse (Figure 4) can be statistically tested by calculating the probability that an overlap of BCR sequences between diagnosis and relapse samples can happen by chance. The hypergeometric test showed that the probability of observing equal or greater BCR sequence overlap between diagnosis and relapse was significantly higher than expected by chance for all pairs tested (*P*-values<10^−15^, Supplementary Table S11). For example, patient A had two unique BCRs with nine nucleotide differences from the central BCR at diagnosis, of which one was resampled at relapse 3 years later. The probability of this occurring by chance if relapse had emerged from a single BCR is negligible. This was confirmed by sequencing diagnostic and relapse samples from a subset of the patients (A, C, D and E) on separate PacBio sequencing runs, and sequences were matched to the B-ALL clones. Indeed, even at a low sequencing depth, overlap of non-central BCR sequences was observed between diagnosis and relapse (Supplementary Table S12 and Supplementary Figure S5), confirming that this observation is independent of sequencing platform. To account for any mutational biases generated through PCR and sequencing, the baseline observed probabilities of overlap of mutations in independent V-(D)-J recombination events were used to compare the diagnosis-relapse samples. There were significantly higher numbers of overlapping mutations between diagnosis and relapse samples than were observed between independent clonal rearrangements (*P*-values <10^−^^50^, Supplementary Table S13).

The potential of overlap originating from Illumina multiplex identification (MID) tag mixing was eliminated as samples from each time point and each technical repeat were sequenced on different MiSeq runs. PCR error can be distinguished from true BCR subclonal mutations as they are subjected to different mutational processes, thus exhibiting different mutational profiles. The relative frequencies of each mutation within mutational triplets were compared between B-ALL clonal sequences and control genes (human β-globin/*GAPDH*) and showed that 29/96 mutational signatures were significantly different (*P*<0.005, Supplementary Figure S6). After accounting for multiple testing (Bonferroni correction), there were no comparisons with significantly higher overlap between mismatched sites than expected by chance (*P*-values<0.005, Supplementary Figure S7 and Supplementary Table S14). This suggests that PCR/sequencing error is primarily a random process and that there were no common error-prone sites in the BCR sequence during these steps. This supports the notion that variation in these clusters reflects true diversity of B-ALL samples.

Although in all seven cases analyzed, a single large clone represented the great majority of BCR sequences, multiple B-ALL cell clones with distinct BCRs also re-emerged at relapse. B-ALL relapse is frequently associated with the acquisition of drug resistance mutations in genes such as *CREBBP*,^48^ *NT5C2*,^49, 50^ *SETD2*^51^ and others^52^ so we propose that the opportunity/likelihood of acquisition of such mutations increases with the number of residual B-ALL cells during remission, which is in turn determined by partial chemoresistance and/or inadequate therapy (Figure 4).

## Discussion

We describe a technical and analytical approach for quantitative detection of MRD and characterization of B-ALL clonal architecture using BCR sequencing. Given *a priori* knowledge of the clonotypic BCR sequence, MRD detection was at least as sensitive as with qPCR-based methods, while BCR sequencing is universally applicable to all cases irrespective of their cargo of oncogenic mutations. BCR sequencing can detect multiple disease subclones such as those in which B-ALL has undergone secondary rearrangements or cases with two independent B-cell malignancies,^8, 36^ and in this study we show an unexpectedly high incidence of secondary rearrangements in all cases studied. Importantly, multiple clinically significant clones carrying distinct *IgHV-DJ* rearrangements were identified in several B-ALLs. Using the BCR stem regions (*IgHD-J*) as molecular barcodes, we reveal the disparate dynamics of cellular clones suggesting differential responses to therapy, different growth rates within different anatomical sites and emergence of novel clones at relapse corresponding with the cytogenetic evolution. Given the abundance of derivative B-ALL clones, and their independent growth trajectories in response to treatment, monitoring all emerging and growing clones can be of clinical importance and can be achieved using BCR sequencing.

As with somatic mutations elsewhere in the genome,^53^ mutations in the BCR reflect B-ALL clonal diversification and can be used to infer phylogenetic relationships between subclones. Unlike subclones characterized by different oncogene mutations, those defined by differences in BCR sequence do not necessarily display distinct biological behaviors but do capture the evolution and phylogenetic history of the clone. In keeping with the accepted paradigm of cancer evolution, the star-like structures of B-ALL BCR repertoires indicate that each leukemia emerged from a single common B-cell ancestor or stem cell. Importantly, we go on to show that B-cell repertoires at diagnosis and relapse overlapped significantly in all seven cases studied here, indicating that much of the population structure of B-ALL is retained during remission. Nevertheless, a single dominant clone/BCR sequence was always identified that was identical to the dominant clone at diagnosis, with other clones remaining much smaller (Figure 4). Previous studies have shown that B-ALL relapse is often driven by relapse-specific chemoresistance mutations in loci such as the *NT5C2* nucleotidase gene,^49, 50^ or mutations in genes like *SETD2*, *CREBBP* and others, which can be present at diagnosis or gained at relapse.^48, 51^ We propose that the persistence of small B-ALL clones, defined by their unique BCR sequence, is a surrogate for partial inherent chemoresistance and/or inadequate initial therapy. This in turn increases the likelihood of acquisition of *bona fide* chemoresistance mutations in at least one of the surviving cells, whose progeny then go on to dominate relapse (Figure 4). This explains recent observations that subclones without identifiable resistance mutations survive initial chemotherapy and acquire resistance mutations in their descendants.^52^ Also, it explains why the level of MRD at early stages of treatment (for example, day 19) is a good predictor of long-term survival^13, 54^ and emphasizes the importance of residual disease as the conduit for chemoresistance-driven relapse. In this context, the higher sensitivity of our method could be exploited to further enhance the ability of early-stage MRD levels to predict long-term prognosis, by differentiating patients with and without very low-level MRD with greater accuracy and at later stages of initial therapy (we detected MRD on day 28 in six of eight cases who went on to relapse).

In summary, we show the broad applicability and exquisite sensitivity of our BCR sequencing and analysis method for MRD monitoring of B-ALL and use this method to reveal clinically relevant features of B-ALL clonal dynamics, including the extent of clonal diversification through both hypermutation and secondary rearrangement, and the first demonstration of the persistence of multiple small B-ALL clones throughout therapy in patients who went on to relapse. This finding puts the spotlight on early aggressive cytoreduction or change in therapy as a means for decreasing the likelihood of chemoresistant relapse.

## Figures and Tables

**Table 1:** B-ALL patient sample information

*Patient ID*	*Fusion gene and other genetic abnormality*	*BCR sequence pattern in diagnostic sample*	*BCR sequence pattern in relapse or MRD sample*	*Disease status*	*BCR sequencing template*
527^a^	*E2A-PBX1*	Monoclonal expansion	Original clone (low level)	No relapse	RNA
859^a^	*ETV6-RUNX1*	Biclonal expansion^b^ (with secondary rearrangements)	Original clone (low level)	No relapse	RNA
1592^a^	*E2A-PBX1*	Monoclonal expansion	Non-clonal	No relapse	RNA
1611^a^	*E2A-PBX1*	Monoclonal expansion	Non-clonal	No relapse	RNA^c^
1703^a^	*ETV6-RUNX1*	Monoclonal expansion^b^	Monoclonal expansion (novel clone)	CNS relapse (+ low-level BM)	RNA^c^
3243^a^	*BCR-ABL*	Monoclonal expansion	Non-clonal	No relapse	RNA
Patient A^d^	*ETV6-RUNX1*	Monoclonal expansion	Monoclonal expansion (diagnostic clone)	BM relapse	DNA
Patient B^d^	Additional *RUNX1*	Biclonal expansion	Monoclonal expansion (of one of diagnostic clones)	BM relapse	DNA
Patient C^d^	*ETV6-RUNX1*	Biclonal expansion^e^	Biclonal expansion (two diagnostic clones)^e^	BM relapse	DNA
Patient D^d^	*TCF3* rearrangement	Monoclonal expansion	Monoclonal expansion (diagnostic clone)	BM relapse	DNA
Patient E^d^	*ETV6-RUNX1*	Biclonal expansion	Monoclonal expansion (diagnostic clone)	BM relapse	DNA
Patient F^d^	*ETV6-RUNX1*	Monoclonal expansion	Biclonal expansion (with diagnostic clones)	Combined relapse	DNA
Patient G^d^	*ETV6-RUNX1;* -X; abn. Chr 3,4,7,12,15	Biclonal expansion	Monoclonal expansion (novel clone)	Combined relapse	DNA
Patient H^d^	*ETV6-RUNX1*, loss of one *CDKN2A* and one *MLL* signal	Polyclonal expansion	Non-clonal	CNS only relapse	DNA
Patient I^d^	Bi-allelic *CDKN2A* deletion	Polyclonal expansion	Monoclonal expansion (of one of diagnostic clones)	BM relapse (+low-level CNS)	DNA

Abbreviations: B-ALL, B-cell acute lymphoblastic leukemia; BCR, B-cell receptor; BM, bone marrow; CNS, central nervous system; MRD, minimal residual disease.

a Treated under the UKALL XI protocol.^55^

b The 'diagnostic' samples from patients 859 and 1703 were taken after treatment had commenced.

c DNA samples were also available from these samples (see Supplementary Figure S2).

d Treated under the UKALL2003 protocol.^55^

e Likely to represent bi-allelic VDJ rearrangements in the transformed B cell.

## Notes

GV receives an unrestricted educational grant from Celgene and is a Consultant for and holds stock in Kymab Ltd. RB-R is a Consultant for VHSquared. MH has performed consultancy for Boehringer-Ingelheim. The remaining authors declare no conflict of interest.
