# Supplementary material for: Eye on the B-ALL: B-cell receptor repertoires reveal persistence of numerous B-lymphoblastic leukemia subclones from diagnosis to relapse
Source: Leukemia. 2016 Jun 17;30(12):2312–21. doi: 10.1038/leu.2016.142 (PMC5155029; doi:10.1038/leu.2016.142)
Supplement: Supplementary Information [file leu2016142x1.doc]

**Eye on the B-ALL: B-cell receptor repertoires reveal persistence of numerous B-lymphoblastic leukemia subclones from diagnosis to relapse**

Rachael J. M. Bashford-Rogers^1,2*^, Katerina A. Nicolaou^3*^, Jack Bartram^4,5^, Nicholas J. Goulden^4^, Loizos Loizou^6^, Laura Koumas^3^, Jianxiang Chi^3^, Mike Hubank^5^, Paul Kellam^1,7^, Paul A. Costeas^3^ and George S. Vassiliou^1,3,8,9^.

^1^Wellcome Trust Sanger Institute, Wellcome Trust Genome Campus, Hinxton, Cambridge, United Kingdom, CB10 1SA, UK;

^2^Department of Medicine, University of Cambridge, Cambridge Biomedical Campus, Wellcome Trust/MRC Building, Hills Road, Cambridge, CB2 0XY, UK;

^3^The Center for the Study of Hematological Malignancies, 15, Nicandrou Papamina Ave., Nicosia 2032, Cyprus;

^4^Department of Haematology, Great Ormond Street Hospital for Children, London, UK.

^5^Genetics and Genomic Medicine Programme, UCL Institute of Child Health, London WC1N 1EH, UK;

^6^Pediatric Oncology/Hematology Clinic, Archbishop Makarios III Hospital, Nicosia, Cyprus;

^7^Research Department of Infection, Division of Infection and Immunity, University College London, Gower Street, London, United Kingdom, WC1E 6BT, UK;

^8^Cambridge Blood and Stem Cell Biobank and Cancer Molecular Diagnosis Laboratory, Cambridge Biomedical Research Centre, Hills Road, Cambridge, CB2 0QQ.

^9^Corresponding author.

^*^These authors contributed equally to this work.

**SUPPLEMENTARY INFORMATION**

**Materials and Methods detailed sequencing and filtering**

MiSeq libraries were prepared using Illumina protocols by adaptor ligation and sequenced using 250bp or 300bp paired-ended MiSeq (Illumina). MiSeq reads were filtered for base quality (median >32) using QUASR and merged if they contained identical overlapping regions of >65bp, or otherwise discarded. Non-immunoglobulin sequences were removed and only reads with significant similarity to reference IgHV genes in the IMGT database by BLAST were retained (<1x10^-10^ E-value). Primer sequences were trimmed from reads and sequences retained for analysis only if both forward and reverse primer sequences were identified and sequence lengths were >240bp. PacBio sequencing was performed on diagnosis and relapse samples from patients A, C, D and E. PacBio libraries were generated using the PacBio® Barcoded Adapters for Multiplex SMRT® Sequencing protocol and sequenced on a PacBio-RS-II using the P6-C4 chemistry/polymerase. Reads were filtered by SMRT and reads mapped to B-ALL clones (within 8bp).

Table S1: Patient and sequencing information on B-ALL samples and time points.

| **Patient ID** | **Sample ID** | **Time since first sample (days)** | **Target/ control transcript ratio** | **Total BCR sequences in sample** | **Target transcript type**** | **Sample source*** | **Largest cluster (% of BCR sequences)** | **% BM blasts** |
| --- | --- | --- | --- | --- | --- | --- | --- | --- |
| 527 | 527_A | 0 | 13.95 | 124,302 | E2A-PBX1 | BM | 43.733 | NA |
| 527 | 527_B | 8 | 0.02 | 270,572 | E2A-PBX1 | BM | 0.696 | NA |
| 527 | 527_C | 15 | 0 | 756,674 | E2A-PBX1 | BM | 0.32 | NA |
| 527 | 527_D | 30 | 0 | 698,592 | E2A-PBX1 | BM | 0.097^§^ | NA |
| 527 | 527_E | 109 | 0 | 2,320,485 | E2A-PBX1 | BM | 6.818^§^ | NA |
| 527 | 527_F | 889 | 0 | 2,301,914 | E2A-PBX1 | BM | 0.58^§^ | NA |
| 859^†^ | 859_A | 0 | 1.66 | 454,071 | ETV6-RUNX1 | PB | (2.81%+ 2.89%) 5.7 ^λ^ | NA |
| 859 | 859_B | 7 | 0.03 | 786,283 | ETV6-RUNX1 | BM | 0.179^§^ | NA |
| 859 | 859_C | 84 | 0 | 737,736 | ETV6-RUNX1 | BM | 0.738^§^ | NA |
| 859 | 859_D | 374 | 0 | 1,929,858 | ETV6-RUNX1 | BM | 2.159^§^ | NA |
| 859 | 859_E | 1241 | 0 | 2,025,955 | ETV6-RUNX1 | BM | 0.219^§^ | NA |
| 1592 | 1592_A | 0 | 34.6 | 259,439 | E2A-PBX1 | BM | 26.6 | NA |
| 1592 | 1592_B | 12 | 12.98 | 264,698 | E2A-PBX1 | BM | 26.105 | NA |
| 1592 | 1592_C | 33 | 0.02 | 216,356 | E2A-PBX1 | BM | 0.192^§^ | NA |
| 1592 | 1592_D | 554 | 0 | 129,923 | E2A-PBX1 | PB | 1.04^§^ | NA |
| 1611 | 1611_A | 0 | 35.04 | 189,634 | E2A-PBX1 | BM | 27.843 | NA |
| 1611 | 1611_B | 12 | 0 | 264,128 | E2A-PBX1 | BM | 0.448^§^ | NA |
| 1611 | 1611_D | 510 | 0 | 284,526 | E2A-PBX1 | BM | 0.175^§^ | NA |
| 1611 | 1611_F | 944 | 0 | 346,134 | E2A-PBX1 | PB | 1.751^§^ | NA |
| 1703 | 1703_A | 0 | 0.12 | 2,972,494 | ETV6-RUNX1 | PB | 0.39^§^ | NA |
| 1703 | 1703_B | 18 | 0 | 2,209,688 | ETV6-RUNX1 | BM | 1.049^§^ | NA |
| 1703 | 1703_C | 336 | 0 | 1,861,228 | ETV6-RUNX1 | BM | 0.131^§^ | NA |
| 1703 | 1703_D | 567 | 0 | 1,475,750 | ETV6-RUNX1 | BM | 0.353^§^ | NA |
| 1703^†^ | 1703_E | 567 | 3.12 | 1,237,270 | ETV6-RUNX1 | CSF | 3.833 | NA |
| 3243 | 3243_A | 0 | 1.75 | 297,165 | BCR-ABL | BM | 10.196 | NA |
| 3243 | 3243_B | 20 | 0.02 | 372,194 | BCR-ABL | BM | 0.194^§^ | NA |
| 3243 | 3243_C | 31 | 0.01 | 340,706 | BCR-ABL | BM | 0.331^§^ | NA |
| 3243 | 3243_D | 56 | 0 | 315,718 | BCR-ABL | BM | 0.32^§^ | NA |
| 3243 | 3243_E | 91 | 0 | 319,850 | BCR-ABL | BM | 0.451^§^ | NA |
| A | 1_A | 0 | NA | 349621 | NA | PB | 83.637 | >99% |
| A | 1_B | 28 | NA | 29571 | NA | BM | 0.683^§^ | <1% |
| A | 1_C | 1095 | NA | 304306 | NA | PB | 87.410 | 73% |
| B | 2_A | 0 | NA | 76023 | NA | BM | 37.129 | 99% |
| B | 2_B | 28 | NA | 40047 | NA | BM | 0.442^§^ | <1% |
| B | 2_C | 2628 | NA | 39039 | NA | BM | 0.315 | 70% |
| C | 3_A | 0 | NA | 613889 | NA | PB | 31.374 | >90% |
| **Patient ID** | **Sample ID** | **Time since first sample (days)** | **Target/ control transcript ratio** | **Total BCR sequences in sample** | **Target transcript type**** | **Sample source*** | **Largest cluster (% of BCR sequences)** | **% BM blasts** |
| C | 3_B | 28 | NA | 33663 | NA | BM | 0.415^§^ | <1% |
| C | 3_C | 1241 | NA | 530178 | NA | PB | 54.264 | >90% |
| D | 5_A | 0 | NA | 276555 | NA | BM | 79.382 | >99% |
| D | 5_B | 28 | NA | 80196 | NA | BM | 0.320^§^ | <1% |
| D | 5_C | 584 | NA | 183902 | NA | BM | 93.041 | 17% |
| E (original diagnosis) | 6_original_diagnosis | -2701 | NA | 517098 | NA | BM | 39.283 | 99% |
| E | 6_A | 0 | NA | 313649 | NA | BM | 76.577 | 69% |
| E | 6_B | 28 | NA | 75788 | NA | BM | 7.0235 | <1% |
| E | 6_C | 584 | NA | 51945 | NA | BM | 52.838 | 36% |
| F | 7_A | 0 | NA | 166938 | NA | BM | 77.903 | 87% |
| F | 7_C | 1423.5 | NA | 243132 | NA | BM | 52.540 | >90% |
| G | 9_A | 0 | NA | 8172 | NA | BM | 45.301 | 88% |
| G | 9_B | 28 | NA | 201909 | NA | BM | 0.185^§^ | <1% |
| G | 9_C | 1022 | NA | 9157 | NA | BM | 2.839 | 97% |
| H | 10_A | 0 | NA | 443404 | NA | BM | 23.162 | 95% |
| H | 10_B | 28 | NA | 41428 | NA | BM | 0.342^§^ | <1% |
| H | 10_C | 839.5 | NA | 307372 | NA | BM | 0.371^§^ | <1% |
| I | 14_A | 0 | NA | 206322 | NA | PB | 43.565 | 70% |
| I | 14_B | 28 | NA | 12274 | NA | BM | 0.725 | <1% |
| I | 14_C | 949 | NA | 7631 | NA | BM | 6.762 | 75% |

Samples highlighted in orange denote the primary samples for each patient. No CNS involvement was detected at primary diagnosis for any patient.

* Abbreviations: BM is bone-marrow, PB is peripheral blood and CSF is cerebrospinal fluid.

** E2A-PBX1: gene fusion between the transcription factor *E2A* with the homeodomain protein *PBX1*. ETV6-RUNX1: gene fusion between the transcription factor *TEL* with the transcription factor *AML1*. BCR-ABL: gene fusion between the “breakpoint cluster region” (*BCR*), a 5.8 kbp region of DNA on chromosome 22 (22q11), with the tyrosine kinase *ABL1*.

† Samples representing the highest tumour burden in time series for patient (denoted “primary sample”), although taken after treatment had commenced in the patients.

^§^ Samples where the maximum cluster does not represent the B-ALL clone.

^λ^ The two largest clusters in the primary sample from patient 859 has two clusters related by secondary rearrangements that total 5.7% of the total repertoire (2.807% + 2.891%).

Table S2: The number of filtered BCR reads and largest cluster sizes (as a percentage of total BCR reads) for the healthy individuals in this study.

| **Patient** | **Number of filtered BCR reads** | **Largest Cluster (%)** |
| --- | --- | --- |
| Healthy 1 | 245,151 | 0.138 |
| Healthy 2 | 113,188 | 0.160 |
| Healthy 3 | 262,198 | 0.162 |
| Healthy 4 | 296,206 | 0.185 |
| Healthy 5 | 274,907 | 0.192 |
| Healthy 6 | 344,331 | 0.217 |
| Healthy 7 | 288,934 | 0.240 |
| Healthy 8 | 270,374 | 0.346 |
| Healthy 9 | 89,766 | 0.358 |
| Healthy 10 | 260,037 | 0.431 |
| Healthy 11 | 280,619 | 0.436 |
| Healthy 12 | 286,428 | 0.618 |
| Healthy 13 | 156,200 | 0.633 |
| Healthy 14 | 231,479 | 0.654 |
| Healthy 15 | 109,301 | 0.694 |
| Healthy 16 | 256,081 | 0.971 |
| Healthy 17 | 261,055 | 1.844 |
| Healthy 18 | 341,180 | 2.577 |

Table S3: Table of the linear gradients and Pearson product-moment correlation coefficients (R^2^ values) between the percentage of B-ALL BCRs matched per sample and qPCR target to control transcript (T/C) ratios

| **Patient ID** | **R^2^ value** |
| --- | --- |
| 527 | 0.9997 |
| 859 | 0.9997 |
| 1611 | 0.9988 |
| 1703 | 0.9986 |
| 3243 | 1.0000 |
| 1592 | 0.8782 |

Table S4: Table of percentages of B-ALL clonotypic BCR sequences in repeated samples.

| **Patient ID** | **qPCR T/C level** | **Time since first sample (days)** | **% of B-ALL sequences** | | **Total reads in sample** | |
| --- | --- | --- | --- | --- | --- | --- |
|  |  |  | **(initial sample)*** | **(re-amplified)**** | **(initial sample)*** | **(re-amplified)**** |
| 527 | 13.951 | 0 | 41.21494 | - | 124302 | - |
| 527 | 0.0197 | 8 | 0.81457 | - | 270572 | - |
| 527 | 0 | 15 | 0.00249 | 0.04261 | 402300 | 354374 |
| 527 | 0 | 30 | 0.00140 | 0.08697 | 355932 | 342660 |
| 527 | 0 | 109 | 0.00000 | 0.00000 | 584090 | 367277 |
| 527 | 0 | 889 | 0.00016 | 0.00180 | 528339 | 389194 |
| 859 | 1.6612 | 0 | 2.89096 | - | 454071 | - |
| 859 | 0.0292 | 7 | 0.21739 | 0.18325 | 431023 | 355260 |
| 859 | 0.0001 | 84 | 0.00159 | 0.00028 | 376848 | 360888 |
| 859 | 0 | 374 | 0.00065 | 0.00032 | 306681 | 310220 |
| 859 | 0 | 1241 | 0.00029 | 0.00031 | 350621 | 322405 |
| 1592 | 34.6048 | 0 | 31.45017 | - | 259439 | - |
| 1592 | 12.9828 | 12 | 27.33152 | - | 264698 | - |
| 1592 | 0.0211 | 33 | 0.24774 | - | 216356 | - |
| 1592 | 0 | 554 | 0.00000 | - | 129923 | - |
| 1611 | 35.0403 | 0 | 26.48259 | - | 189634 | - |
| 1611 | 0.0013 | 12 | 0.90122 | 0.18099 | 11410 | 249814 |
| 1611 | 0 | 19 | 0.06560 | 0.00000 | 7678 | 249451 |
| 1611 | 0 | 33 | 0.00000 | 0.00000 | 1258 | 282810 |
| 1611 | 0 | 510 | 0.00000 | - | 128213 | - |
| 1611 | 0 | 944 | 0.00000 | - | 346134 | - |
| 1703 | 0.1211 | 0 | 0.00266 | 0.13930 | 84544 | 455790 |
| 1703 | 0 | 18 | 0.00005 | 0.00527 | 315140 | 364656 |
| 1703 | 0 | 336 | 0.00000 | 0.00000 | 83272 | 351070 |
| 1703 | 0.0002 | 567 (blood) | 0.00033 | 0.00027 | 85529 | 269857 |
| 1703 | 3.1218 | 567 (CSF) | 3.38261 | - | 457825 | - |
| 3243 | 1.7453 | 0 | 10.73040 | - | 297165 | - |
| 3243 | 0.0219 | 20 | 0.08141 | - | 372194 | - |
| 3243 | 0.0102 | 31 | 0.02319 | - | 340706 | - |
| 3243 | 0.0006 | 56 | 0.00063 | - | 315718 | - |
| 3243 | 0 | 91 | 0.00000 | - | 319850 | - |

* The initial BCR sequencing dataset.

** RNA was re-amplified and sequenced independently.

**Table S5: False positive rate for detecting B-ALL MRD by matching of B-ALL BCR sequences in unrelated BCR datasets derived from 8 healthy individuals.**

| **B-ALL patient** | **Number of unrelated healthy BCRs tested against B-ALL cluster** | **Number of reads matched** |
| --- | --- | --- |
| **B-ALL 1** | 3,730,269 | 0 |
| **B-ALL 2** | 4,098,690 | 0 |
| **B-ALL 3** | 4,097,093 | 0 |
| **B-ALL 4** | 3,836,054 | 0 |
| **B-ALL 5** | 3,922,068 | 1 |
| **B-ALL 6** | 3,796,487 | 0 |
| **Total** | 23,480,661  (2,720,172 unique sequences) | 1 |

* To determine the false positive-rate for B-ALL BCR sequence detection (i.e. the chance for the same IgH V-DJ rearranged sequence to be generated in independent B-cell clones by chance), we used MRDARCY to search for the B-ALL BCR sequences from 6 B-ALL patients in unrelated sequencing datasets from 8 unselected healthy individuals using the same parameters. A total of 23,480,661 BCR sequences were tested, with only a single read matching to a B-ALL BCR cluster sequence in patient 1611. This sequence was unmutated with short non-template additions (4bp) and was 100% identical to a minor BCR clone in the patient D B-ALL cluster (representing 0.0008% in healthy 1 and 0.0633% in diagnostic patient 1611 sample). Therefore the presence of unrelated sequences matching the B-ALL-specific BCR sequence by chance occurred at a rate of 1 in 2,720,172 unique BCR sequences/cells.

**Table S6: Linear regression parameter fitting to dilution series (from Figure 1D) to estimate increase in sensitivity between multiplex and singleplex PCR amplification.**

| **Dilution series** | **R^2^-value*** | **Intercept*** | **Gradient*** |
| --- | --- | --- | --- |
| **Multiplex dilution** | 0.9680 | 6.5066 | -1.5885 |
| **Singleplex dilution** | 0.9828 | 9.1143 | -1.5961 |

*Linear regression parameters fitted to log(proportion of reads clone corresponding to B-ALL clone) versus log(dilution), where e^-(difference between intercepts)^ corresponds to the mean increase in sensitivity between multiplex and singleplex strategies.

**Table S7:** qPCR and BCR sequencing detection of B-ALL in CSF relapse sample.

| **Source*** | **Target/control transcript level**** | **% B-ALL BCR reads (from RNA)** | **% B-ALL BCR reads (from DNA)** |
| --- | --- | --- | --- |
| Day 0, PB | 0.121 | 0.00266 | 28.63019 |
| Day 18, BM | 0 | 5.42E-05 | 0.804093 |
| Day 336, BM | 0 | 0 | - |
| Day 567, BM | 0.000222 | 0.000332 | - |
| Day 567, CSF | 3.122 | 3.38 | - |

* Abbreviations: BM is bone-marrow, PB is peripheral blood and CSF is cerebrospinal fluid.

** Target transcript: TEL/AML1 translocation.

**Table S8: Table of the properties of the largest two clusters in patient 859.**

|  | **Cluster 1** | **Cluster 2** |
| --- | --- | --- |
| **Cluster size (% of total sequences)** | 2.469 | 2.379 |
| **N reads** | 11211 | 10801 |
| **Number of unique sequences in cluster** | 2858 | 2037 |
| **IgHV gene** | IGHV4-34*01 | IGHV1-2*02 |
| **IgHJ gene** | IGHJ6*03 | IGHJ6*03 |
| **Number of sequences representing most frequently observed BCR** | 5625 | 6603 |
| **Mean distance from most frequently observed BCR** | 2.281 | 2.135 |

**Table S9: Table of stem sequences involved in B-ALL secondary rearrangements.**

**Table S10. Enrichment of AID mutational motifs within the B-AL clusters.**

| **Patient ID** | **P-value*** | **Number of sequences analysed** |
| --- | --- | --- |
| Patient 1592 | <1e-8 | 1128 |
| Patient 1611 | <1e-8 | 1492 |
| Patient 1703 | <1e-8 | 584 |
| Patient 3243 | <1e-8 | 592 |
| Patient 527 | <1e-8 | 1262 |
| Patient 859 | <1e-8 | 492 |
| Patient A | <1e-8 | 2468 |
| Patient B | <1e-8 | 759 |
| Patient C | <1e-8 | 2516 |
| Patient D | <1e-8 | 3180 |
| Patient E | <1e-8 | 2596 |
| Patient G | <1e-8 | 104 |
| Patient H | <1e-8 | 1370 |
| Patient I | <1e-8 | 1105 |

* Using the Poisson test on the distribution of mutations away from the central BCR in the B-ALL cluster that are within AID/UNG mutational motifs (WRCY/ RGYW or WA/TW, where W=adenine or thymine, R=purine, C=cytosine, Y=pyrimidine, and the target base is underlined).

**Table S11. Probabilities of BCR repertoire overlap between diagnostic and relapse samples occurring by chance.**

| **Cluster ID** | **Distance from central BCR** | **BCRs observed at Time A** | **BCRs observed at both time points** | **BCRs observed at Time C** | **P-value of observed overlap*** |
| --- | --- | --- | --- | --- | --- |
| Patient A: Cluster 1 | 1 | 633 | 632 | 632 | 1 |
| Patient A: Cluster 1 | 2 | 1816 | 1689 | 1901 | <10E-15 |
| Patient A: Cluster 1 | 3 | 101 | 87 | 101 | <10E-15 |
| Patient A: Cluster 1 | 4 | 2 | 2 | 4 | <10E-15 |
| Patient A: Cluster 1 | 5 | 1 | 0 | 0 | NA |
| Patient A: Cluster 1 | 6 | 4 | 1 | 1 | <10E-15 |
| Patient A: Cluster 1 | 7 | 2 | 1 | 1 | <10E-15 |
| Patient A: Cluster 1 | 8 | 2 | 1 | 1 | <10E-15 |
| Patient A: Cluster 1 | 9 | 2 | 1 | 1 | <10E-15 |
| Patient C: Cluster 1 | 1 | 692 | 690 | 690 | 1 |
| Patient C: Cluster 1 | 2 | 1576 | 1461 | 1598 | <10E-15 |
| Patient C: Cluster 1 | 3 | 52 | 47 | 55 | <10E-15 |
| Patient C: Cluster 1 | 4 | 3 | 1 | 1 | 3.55E-15 |
| Patient C: Cluster 1 | 5 | 1 | 1 | 1 | <10E-15 |
| Patient C: Cluster 1 | 6 | 3 | 2 | 2 | <10E-15 |
| Patient C: Cluster 1 | 7 | 2 | 2 | 2 | <10E-15 |
| Patient C: Cluster 1 | 8 | 2 | 2 | 2 | <10E-15 |
| Patient C: Cluster 1 | 9 | 6 | 5 | 6 | <10E-15 |
| Patient C: Cluster 1 | 10 | 3 | 2 | 3 | <10E-15 |
| Patient C: Cluster 1 | 11 | 33 | 32 | 34 | <10E-15 |
| Patient C: Cluster 2 | 1 | 742 | 742 | 742 | 1 |
| Patient C: Cluster 2 | 2 | 1777 | 1663 | 1961 | <10E-15 |
| Patient C: Cluster 2 | 3 | 63 | 52 | 62 | <10E-15 |
| Patient C: Cluster 2 | 4 | 4 | 3 | 4 | <10E-15 |
| Patient C: Cluster 2 | 5 | 5 | 3 | 3 | <10E-15 |
| Patient C: Cluster 2 | 6 | 5 | 5 | 5 | <10E-15 |
| Patient C: Cluster 2 | 7 | 3 | 2 | 2 | <10E-15 |
| Patient C: Cluster 2 | 8 | 3 | 3 | 3 | <10E-15 |
| Patient C: Cluster 2 | 9 | 5 | 5 | 5 | <10E-15 |
| Patient C: Cluster 2 | 10 | 3 | 3 | 3 | <10E-15 |
| Patient C: Cluster 2 | 11 | 37 | 32 | 40 | <10E-15 |
| Patient C: Cluster 2 | 12 | 3 | 2 | 3 | <10E-15 |
| Patient D: Cluster 1 | 1 | 771 | 767 | 768 | 1 |
| Patient D: Cluster 1 | 2 | 1189 | 1069 | 1240 | <10E-15 |
| Patient D: Cluster 1 | 3 | 40 | 30 | 44 | <10E-15 |
| Patient D: Cluster 1 | 4 | 1 | 0 | 0 | NA |
| Patient D: Cluster 1 | 5 | 1 | 0 | 0 | NA |
| Patient D: Cluster 1 | 6 | 4 | 0 | 0 | NA |
| Patient D: Cluster 1 | 7 | 5 | 3 | 3 | <10E-15 |
| Patient D: Cluster 1 | 8 | 8 | 7 | 7 | <10E-15 |
| Patient D: Cluster 1 | 9 | 6 | 4 | 4 | <10E-15 |
| Patient D: Cluster 1 | 10 | 4 | 2 | 2 | <10E-15 |
| Patient D: Cluster 1 | 11 | 6 | 1 | 1 | <10E-15 |

| **Cluster ID** | **Distance from central BCR** | **BCRs observed at Time A** | **BCRs observed at both time points** | **BCRs observed at Time C** | **P-value of observed overlap*** |
| --- | --- | --- | --- | --- | --- |
| Patient E: Cluster 1 | 1 | 726 | 624 | 624 | 1 |
| Patient E: Cluster 1 | 2 | 844 | 391 | 393 | <10E-15 |
| Patient E: Cluster 1 | 3 | 36 | 17 | 18 | <10E-15 |
| Patient E: Cluster 1 | 4 | 3 | 3 | 3 | <10E-15 |
| Patient E: Cluster 1 | 5 | 4 | 3 | 4 | <10E-15 |
| Patient E: Cluster 1 | 6 | 2 | 2 | 2 | <10E-15 |
| Patient E: Cluster 1 | 7 | 2 | 2 | 3 | <10E-15 |
| Patient E: Cluster 1 | 8 | 0 | 0 | 1 | NA |
| Patient E: Cluster 1 | 9 | 2 | 2 | 3 | <10E-15 |
| Patient E: Cluster 1 | 10 | 1 | 1 | 1 | <10E-15 |
| Patient F: Cluster 1 | 1 | 765 | 755 | 759 | 1 |
| Patient F: Cluster 1 | 2 | 594 | 565 | 753 | <10E-15 |
| Patient F: Cluster 1 | 3 | 38 | 36 | 41 | <10E-15 |
| Patient F: Cluster 1 | 4 | 4 | 4 | 4 | <10E-15 |
| Patient F: Cluster 1 | 5 | 8 | 7 | 7 | <10E-15 |
| Patient F: Cluster 1 | 6 | 4 | 4 | 5 | <10E-15 |
| Patient F: Cluster 1 | 7 | 8 | 8 | 10 | <10E-15 |
| Patient F: Cluster 1 | 8 | 14 | 12 | 12 | <10E-15 |
| Patient F: Cluster 1 | 9 | 7 | 6 | 8 | <10E-15 |
| Patient F: Cluster 1 | 10 | 8 | 6 | 9 | <10E-15 |
| Patient F: Cluster 1 | 11 | 7 | 7 | 16 | <10E-15 |
| Patient F: Cluster 1 | 12 | 6 | 3 | 3 | <10E-15 |
| **Cluster ID** | **Distance from central BCR** | **BCRs observed at day 0** | **BCRs observed at both time points** | **BCRs observed at day 567** | **P-value of observed overlap*** |
| Patient 1705: Cluster 1 | 1 | 501 | 490 | 828 | <10E-15 |
| Patient 1705: Cluster 1 | 2 | 352 | 37 | 2688 | <10E-15 |
| Patient 1705: Cluster 1 | 3 | 130 | 3 | 1312 | 3.34E-10 |
| Patient 1705: Cluster 1 | 4 | 70 | 1 | 683 | 1.54E-06 |
| Patient 1705: Cluster 1 | 5 | 13 | 1 | 443 | 1.01E-09 |
| Patient 1705: Cluster 1 | 6 | 8 | 1 | 405 | 3.71E-12 |
| Patient 1705: Cluster 1 | 7 | 7 | 1 | 372 | 2.29E-14 |

* P-value of observed overlap occurring by chance under the null hypothesis that the relapse clone emerged from a single B-cell clone exhibiting a single BCR sequence.

Table S12. Summary of overlap between samples sequenced by PacBio.

|  | **Total PacBio Sequences** | |  | **Number of shared sequences** | |
| --- | --- | --- | --- | --- | --- |
| **Cluster ID** | **Time 1** | **Time 2** |  | **Unique** | **Total** |
| **Patient A: Cluster 1** | 129 | 1 |  | 1 | 3 |
| **Patient C: Cluster 2** | 42 | 2 |  | 0 | 0 |
| **Patient D: Cluster 1** | 8 | 9 |  | 1 | 3 |
| **Patient E: Cluster 1** | 5 | 6 |  | 1 | 2 |

Table S13. Statistical information about overlapping mutations.

| **Sample** | **Mean probability of overlapping mutations** | **P-value** | **Number of comparisons** |
| --- | --- | --- | --- |
| **Healthy V gene independent mutations** | 0.02064 | 0.50000 | 945405 |
| **Patient 1703 (RNA+DNA)*** | 0.48415 | <10^-50^ | 1388 |
| **Patient A** | 0.27484 | <10^-50^ | 1288 |
| **Patient C (Cluster 1)** | 0.22457 | <10^-50^ | 1514 |
| **Patient C (Cluster 2)** | 0.23011 | <10^-50^ | 1408 |
| **Patient D** | 0.21639 | <10^-50^ | 1562 |
| **Patient E** | 0.31063 | <10^-50^ | 1468 |
| **Patient F** | 0.10897 | <10^-50^ | 1560 |

* Patient 1705 cluster 1 (day 0 (combined RNA and DNA sequencing datasets) against relapse (day 567, RNA sequencing dataset)).

Table S14. Summary of overlap between sites in erroneous sequences in control genes.

|  | **Number of sequence comparisons** | **% of comparisons with any overlap between erroneous sites** | **% of comparisons with significant overlap between erroneous sites** | **Length of sequence (bp)** |
| --- | --- | --- | --- | --- |
| **GAPDH** | 60000 | 0.205% | 0% | 160 |
| **Beta-globin** | 10000 | 1.278% | 0% | 334 |

**Figure S1. Reproducibility of B-ALL BCR repertoires.**

Correlation of network parameters for patients A-I between two independent amplification and sequencing rounds from DNA, with at least 2000 reads per sample for **A)** vertex Gini indices, **B)** cluster Gini indices and **C)** maximum cluster sizes. The R^2^-values and linear equations are given in the bottom of each plot.

Figure S2. Detection of B-ALL BCR sequences in RNA and DNA samples. Bar-chart showing the percentages of B-ALL sequences from BCR datasets generated from either the RNA or DNA from B-ALL patients (red and blue bars respectively). The BCR RNA expression in mature B-cells is greater than that of pre-B-cells or immature B-cells [^17^](#_ENREF_17). To test the possibility that BCR expression in B-ALL cells/samples may be lower than in non-malignant B-cells, the DNA and RNA BCR repertoires were compared in three patient samples. For every patient time point, B-ALL-derived BCR sequences were detected in the DNA sample at a much higher percentages of total BCR sequences compared to the percentage derived from studying the matched RNA sample. Therefore, although BCR sequencing is highly sensitive for the detection of B-ALL-derived sequences, the RNA BCR repertoire may be significantly underestimating the true percentage of B-ALL cells in the sample and the use of DNA repertoires in B-ALL may further increase the sensitivity for MRD detection.

Figure S3. High-throughput detection of secondary rearrangements in all B-ALL patient samples. The percentages of BCR sequences containing the corresponding stem sequences from the major clones in each patient were identified in serial time points (encompassing the IgHD-IgHJ region and non-template additions up to 3bp 3’ to the end of the IgHV gene, Table S9). The different IgHV gene usages are plotted in different colours, and the highest three observed IgHV genes are indicated above each plot. The grey boxes indicate the 99^th^ percentile frequency detection rate of each stem sequence in healthy individuals.

**Figure S4.** Comparison of the numbers of mutations within the CDR1-3 or FWR1-3 regions of each BCR within the B-ALL clusters was determined using IMGT annotation and normalized for sequence length.


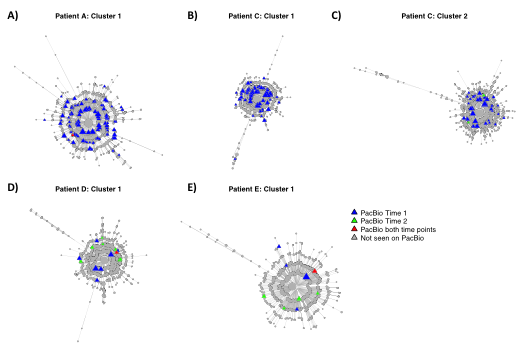


**Figure S5. Overlap of B-ALL clonal sequences between diagnosis and relapse sequenced on PacBio for A) Patient 1 (cluster 1), B) Patient 3 (cluster 1), C) Patient 3 (cluster 2), D) Patients 5 (cluster 1) and E) Patient 6 (cluster1).** Unrooted maximum parsimony trees generated from the MiSeq sequencing showing the relationships between sequences observed in diagnostic and relapse where branch lengths are proportional to the number of varying bases (evolutionary distance) (as in Figure 3B). Tips represent BCR sequences with point sizes correlating with the proportion of reads for a particular sequence (for display purposes this is not the case for the central cluster, whose size is fixed). Overlaid onto the trees are highlighted the tree tips if they were observed in the PacBio datasets at diagnosis (Time 1, blue), relapse (Time 2, green), both time points (red), or not observed by PacBio (grey). Bootstrapping was performed to evaluate the reproducibility of the trees suggesting strong support for the majority of the branches (>70% certainty for branches).


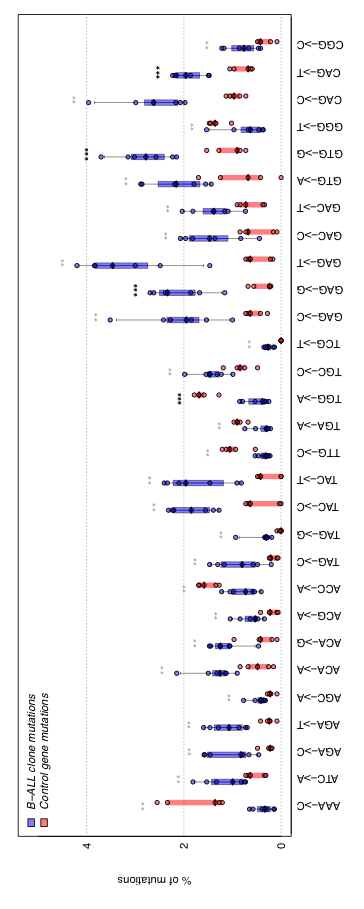


**Figure S6. Comparison of mutational profiles of B-ALL and control genes clusters.** To quantify the impact of PCR/sequencing error, mutations away from the dominant sequences within clusters in B-ALL or control gene datasets were quantified and classified in terms of the targeted base along with the immediate upstream and downstream bases (triplet). The relatively frequencies of each mutation within the mutational triplet was compared between the B-ALL clones and control genes (human β-globin and human GAPDH). The figure shows boxplots of the frequencies of each mutation that was significantly different between the two groups (p<0.005 denoted **, 29 out of a total of 96 mutational signatures were significantly different and 4 out of a total of 96 mutational signatures were significantly different after multiple testing correction, p< 5.21x10^-5^ denoted ***).

**Figure S7. Strategy for determining if there is an enrichment of PCR/sequencing error at particular positions. A)** An example of alignments of erroneous sequences to the gene consensus sequence (performed on GAPDH), and the corresponding positions of errors within these alignments for independently amplified samples, and **B)** the corresponding number of overlapping erroneous sites between independently samples. From this, the probability of random overlap between the sites of PCR/sequencing error were calculated using the hypergeometric distribution.
